# The Effects of an Eight over Cricket Bowling Spell upon Pace Bowling Biomechanics and Performance within Different Delivery Lengths

**DOI:** 10.3390/sports7090200

**Published:** 2019-08-30

**Authors:** Samuel J. Callaghan, Robert G. Lockie, Warren A. Andrews, Walter Yu, Robert F. Chipchase, Sophia Nimphius

**Affiliations:** 1 Centre for Exercise and Sports Science Research, School of Medical and Health Sciences, Edith Cowan University, Joondalup, Western Australia 6027, Australia; 2 High Performance Department, Western Australian Cricket Association, Perth, Western Australia 6004, Australia; 3 Department of Kinesiology, California State University, Fullerton, CA 92831, USA; 4 Sports Performance Research Institute New Zealand, University of Technology, Auckland 1010, New Zealand

**Keywords:** fast bowler, ball release speed, statistical parametrical mapping, load monitoring, fatigue

## Abstract

Pace bowlers must often perform extended bowling spells with maximal ball release speed (BRS) while targeting different delivery lengths when playing a multi-day match. This study investigated the effect of an eight over spell upon pace bowling biomechanics and performance at different delivery lengths. Nine male bowlers (age = 18.8 ± 1.7 years) completed an eight over spell, while targeting different lengths (short: 7–10 m, good: 4–7 m, full: 0–4 m from the batter’s stumps, respectively) in a randomized order. Trunk, knee and shoulder kinematics and ground reaction forces at front foot contact (FFC), as well as run-up velocity and BRS were measured. Paired sample *t*-tests (*p* ≤ 0.01), Hedges’ *g* effect sizes, and statistical parametrical mapping were used to assess differences between mean variables from the first and last three overs. No significant differences (*p* = 0.05–0.98) were found in any discrete or continuous variables, with the magnitude of difference being trivial-to-medium (g = 0.00–0.73) across all variables. Results suggest pace bowlers sustain BRS through a single eight over spell while tolerating the repeatedly high whole-body biomechanical loads as suggested by maintaining the kinematics or technique at the assessed joints during FFC. Practically, the findings are advantageous for bowling performance and support current bowling load monitoring practices.

## 1. Introduction

Cricket has a number of different match formats, which range in length from a few hours (e.g., twenty20 and one-day cricket) to several days (e.g., multi-day matches). Due to the length of the match, the multi-day format dictates a greater total workload (i.e., the combination of internal and external sport stressors) for all players [1]. Specifically, for pace bowlers, the increased total workload, is largely attributed to the greater number of deliveries bowled, often within an extended bowling spell. Despite the use of extended bowling spells within the longer match formats, pace bowlers are generally expected to be able to maintain a high ball release speed (BRS) each delivery. An increased BRS may disrupt the timing and stroke execution of an opposing batter, increasing the likelihood of dismissal. Pace bowlers will also employ various delivery lengths (i.e., short, good and full), defined as the distance the ball lands from the batter [2], to further assist with dismissing batters. 

Within the multi-day match format, it is not uncommon for a single bowling spell to last up to eight overs [3]. Subsequently, previous scientific investigations have investigated the implications of a single eight-over [3,4], 10-over [5], four-three-three over [6] and two-by-six over [7,8] bowling spells upon BRS among amateur, state first grade, high performance, elite and junior pace bowlers, respectively. No significant differences in BRS were reported for any study [3,4,5,6,7,8]. Regardless of the level of competition or age, pace bowlers should be able to maintain BRS during a single, two or three extended bowling spells. However, the influence of an extended bowling spell upon the underlying biomechanical factors (i.e., vertical and braking ground reaction force (GRF), braking impulse and shoulder angle at ball release) which have previously been associated with BRS requires further investigation [9,10,11]. Further to this, there has been no research to date that has assessed the implications of different delivery lengths upon BRS throughout an extended bowling spell, despite this being common practice within match-play.

To date, there has been a limited amount of research which has investigated any potential changes to pace bowling technique that underpin performance during an extended bowling spell [3,4,5,7]. Of the research which has been conducted, Portus, Sinclair, Burke, Moore, and Farhart [3], Crewe, Campbell, Elliott and Alderson [4] and Schaefer, O’Dwyer, Ferdinands and Edwards [5] have all reported non-significant changes in kinematics and BRS in state first grade and junior pace bowlers. Furthermore, Crewe, Campbell, Elliott and Alderson [4] and Schaefer, O’Dwyer, Ferdinands and Edwards [5] also reported no significant changes in GRF measures (i.e., peak GRF, time to peak GRF, GRF impulse, and GRF loading rate) during front foot contact (FFC) in either the vertical or horizontal planes among junior pace bowlers. With respect to GRF, additional research is still required to determine whether young adult pace bowlers, who typically produce higher relative and absolute GRF during FFC [10,12,13], are also able to maintain FFC GRFs throughout an extended bowling spell. If changes in a pace bowler’s FFC GRFs, as a representation of whole-body external biomechanical load, are present, this may necessitate alterations in bowling technique, so as to counter contradictory changes in other areas of the pace bowling action [10].

Potential changes in GRF during FFC are of practical importance when considering current pace bowling load management practices. One of the most common and practical means of assessing pace bowling loads, regardless of competition level, is counting the total number of delivers bowled. This external measure of load implies that the physical demands of each delivery are consistent, despite match-play dictating changes in delivery lengths. Furthermore, no consideration is given to the length of spell or stage of a match (i.e., end of the fifth day of a test match) and how this might affect the GRFs experienced by a pace bowler. This is important, as Stronach, et al. [14] suggested that the repeated bouts of eccentric contractions associated with pace bowling during extended bowling spells may lead to high levels of fatigue. A perspective supported by Pote et al. [6] who reported a significant increase in local (i.e., lower-limb) rating of perceived exertion between the first four and last six overs of a four-three-three bowling spell among young adult (17–21 years of age) pace bowlers. Therefore, additional research is required to determine the implications of an extended bowling spell targeting different delivery lengths upon the whole-body biomechanical load, as represented by FFC GRFs, for young adult pace bowlers. Any changes in biomechanical load throughout a spell are of practical importance to strength and conditioning coaches or sport scientists as they will have implications upon pace bowling loads which are critical to the well-being and performance of pace bowlers [15].

There is a need to quantify the interaction of biomechanical variables which maintain BRS during an extended bowling spell, with respect to different delivery lengths (i.e., short, good and full). This will provide valuable information about whether a pace bowler’s technique could change during bowling spells in the multi-day match format, and whether this could influence current load management practices. Therefore, this research determined if there were any biomechanical and performance changes between the first three overs and last three overs of an eight over spell with respect to short, good and full delivery lengths during FFC in pace bowlers. It was hypothesized that GRF will differ between the first and last three overs of an eight over spell, but there will be no changes in pace bowling kinematics, with respect to different delivery lengths. It was further hypothesized that BRS will remain constant throughout the eight over spell for each delivery length.

## 2. Materials and Methods

### 2.1. Participants

A convenience sample of nine healthy male cricketers was recruited for this study (age = 18.8 ± 1.7 years, height = 1.9 ± 0.1 m, weight = 80.7 ± 9.5 kg). Inclusion criteria required current or previous involvement in an Australian state cricket development pathway or state premier grade cricket, greater than 17 years of age, and free from any existing medical conditions that would be contradictory to participation. The sample included six right-arm and three left-arm pace bowlers. All participants, and where appropriate, guardians of participants under 18 years of age, received a clear explanation of the study, including the risks and benefits of participation, and provided written informed consent prior to participation. The research was approved by the Edith Cowan University Ethical Review Board (Approval 11948) and conformed to the policy statement with respect to the Declaration of Helsinki.

### 2.2. Procedures

A cross-sectional study design involving a single testing session was utilized to assess the biomechanical and performance differences within short, good, and full delivery lengths during a simulated eight over bowling spell among pace bowlers. The pace bowling assessment incorporated three-dimensional (3D) motion capture and in-ground tri-axial force plates to determine kinematic and kinetic discrete and continuous variables during FFC of the delivery stride. BRS was also recorded as a measure of performance. Mean comparisons between variables from the first and last three overs were used for analysis. Prior to the commencement of data collection, the participant’s age, height, body mass, and anthropometric data were recorded. Anthropometric measurements were necessary for the motion capture analysis, which will be detailed later. A standardized warm-up, consisting of jogging, dynamic stretching of the upper and lower limbs, and progressive speed runs, was used for all participants.

### 2.3. Pace Bowling Performance Test

**Figure 1:**
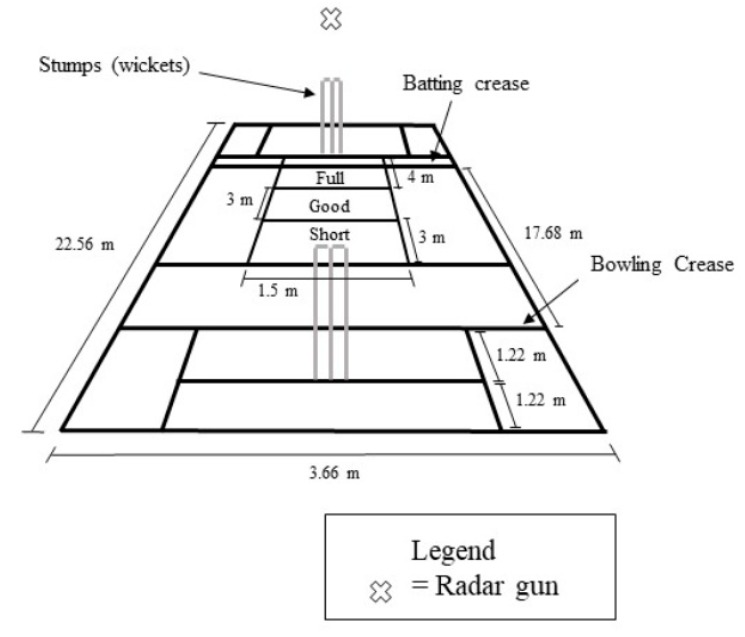
The dimensions of the cricket pitch used during testing. Delivery zones of short (7–10 m from the batter’s stumps), good (4–7 m from the batter’s stumps) and full (0–4 m from the batter’s stumps) are shown.

An eight over spell, comprising 48 maximum effort deliveries of either a short, good, or full length (Figure 1) were performed by each participant [2,3,16,17]. This length of bowling spell is not uncommon within high level cricket and has also been used in previous research for the assessment of kinematic changes in pace bowling technique [3]. The dimensions of the laboratory allowed each participant to use their normal, full length run-up and follow-through, while bowling deliveries on the equivalent of a standard-sized cricket pitch. A four-minute active rest period was provided between each over to comply with match regulations [3,6,17], however this is atypical of match-play as participants were not required to undertake fielding actions. The justification for the use of an active recovery, exclusive of fielding movement patterns is based upon the suggestions of Stronach, Cronin, and Portus [14]. Stronach, Cronin, and Portus [14] outlined that it is the repeated bouts of eccentric contractions associated with the delivery stride of pace bowling during an extended bowling spell which will lead to high levels of fatigue, not low-level intermittent bouts of running. The classification of the various delivery lengths was based upon match and training analysis of pace bowling effectiveness from a state squad competing within Australian national competitions. Assessment protocols routinely used by Cricket Australia also require bowlers to target delivery lengths of short, good, and full [8,18]. Each delivery length was targeted six times during the first and last three overs of the eight over spell in a randomized order. Delivery length was visually identified by two researchers [8]. If a delivery landed on the line between two target lengths, the delivery was classified as of hitting the intended length [3]. Although infrequent, if a participant failed to land the delivery in the intended length, the order of deliveries was revised to ensure the required number of different deliveries was attained. All bowlers used a red Kookaburra four-piece (156 grams) cricket ball (A.G. Thompson Pty. Ltd., Melbourne, Australia) and wore their own bowling spikes during testing. A Stalker Pro ΙΙ speed radar gun (Stalker Radar, Richardson, TX, USA) was located behind the batting stumps net and aimed at the ball release point to measure BRS.

### 2.4. Kinematic Data Collection and Analysis

All trials were recorded using the XSENS motion analysis system (MVN system, XSENS Technology, Enschede, Netherlands) to determine pace bowling kinematics. Following the warm-up and prior to testing, each participant was fitted within a lycra suit (XSENS Technology, Enschede, Netherlands), which housed 17 inertial sensors (0.038 × 0.053 × 0.021 m, 0.03 kg) with a sample frequency of 120 Hz. The inertial sensors were located on the participant’s pelvis, sternum, head and left and right shoulders, upper arms, forearms, hands, upper legs, lower legs, and feet. The inertial sensors (XSENS Technology, Enschede, Netherlands)) consisted of a 3D accelerometer, gyroscope, and magnetometer. Each body segment’s position and orientation can be estimated by integrating the gyroscope and double integrating the accelerometer data, in combination with a biomechanical model [19]. The XSENS motion analysis system has previously been found to be a reliable and valid means of assessing joint angles in dynamic movements [19,20,21]. Additional lengths were also determined between sensor locations and landmarks once the participant was fitted within the lycra suit. A static capture was undertaken prior to pace bowling performance testing and required participants to adopt a stationary N-pose, as per the manufacturer’s recommendations. Following the fitting of the lycra suit and calibration procedures, participants then bowled as many deliveries as required to become familiar with the test environment. 

Data collected from the XSENS motion capture system was exported to Visual3D (Version 6.00.18; C-Motion, Inc., Germantown, MD, USA) for filtering and kinematic analysis. A full-body model was recreated in Visual3D based upon previous cricket pace bowling models [11,22] using the exported 64 virtual marker set that is calculated by the manufacturer software (MVN Studio Version 3.5.3, XSENS Technology, Enschede, The Netherlands) using the measured segments orientations and body dimensions. A second order low pass Butterworth filter with a cut-off frequency of 6 Hz, as determined by a fast Fourier transformation, was applied to all kinematic data [23,24]. Segment orientation was such that the z-axis pointed upward along the longitudinal axis, the x-axis to the participant’s right, and the y-axis forward. Similarly, the global coordinate system was defined with the y-axis pointing down the wicket (towards the batter), the x-axis to the right and the z-axis representing the upwards vertical. Joint angles were calculated as Cardan angles with an axis order rotation of XYZ [11]. Five discrete kinematic parameters were calculated for each trial: Horizontal run-up velocity, front knee angle at FFC and ball release, trunk flexion from FFC to ball release, and bowling arm shoulder angle at ball release. Horizontal run-up velocity in the global frame was determined as per Worthington, King, and Ranson [11]. Front knee angle was calculated as the relative angle between the thigh and shank (full extension = 0°, flexed > 0°). Trunk angle was relative to the global coordinate system (neutral spine position = 0°, flexed > 0°). Shoulder angle was defined as relative to the trunk (anatomical position = 0°) [9,11]. Due to the unique actions of the shoulder during pace bowling, which require full circumduction of the humerus with varying degrees of internal and external rotation, current methods of joint angle calculation are not well defined for the entire motion, leading to errors during calculations [25]. Therefore, while not customary, a separate axis order of rotation (ZYZ) was used to determine shoulder joint angle at the instance of ball release [25]. The use of a separate axis order rotation was undertaken such that an appropriate representation, which is comparative to previous research [9,11] could be provided for the shoulder joint angle variable. The continuous joint-time curve of the front knee from FFC to ball release was also assessed for each delivery. A customized MATLAB (R2015b, The MathWorks Inc, Natick, MA, USA) script was used to time normalize the data to 100% of the stance phase from FFC to ball release using a spline fill pattern prior to statistical analysis of continuous variables using statistical parametric mapping (SPM). 

### 2.5. Kinetic Data Collection and Analysis

An in-ground tri-axial force plate (9287CA, Kistler Group, Winterthur, Switzerland) sampling at 960 Hz was used to collect GRF data during FFC of the delivery stride. FFC corresponded to the first instance at which the vertical GRF exceeded 20 N [26]. Both the XSENS motion capture system and the in-ground force plate were time synchronized through an analog board (Kistler Group, Winterthur, Switzerland) which allowed the XSENS recording software to trigger data capture within the force plate software (Bioware Version 5.3.0.7, Kistler Group, Winterthur, Switzerland) via a voltage rising edge configuration. All trials were filmed with a high-speed video camera (Apple Inc, Cupertino, CA, USA) at 240 Hz from a position perpendicular to the delivery stride to sync FFC on the force plate and ball release using video analysis software (Kinovea–0.8.15, Kinovea, Bordeaux, France) [3,27]. Flooring surface (Mondo S.p.A., Alba, Italy) of the laboratory and on-top of the force platform was consistent. 

Discrete kinetic variables were all measured from FFC to ball release, and included peak vertical and braking forces, vertical and braking impulses, and average vertical and braking loading rates (peak force divided by time from FFC to peak force). The force platform software (Bioware Version 5.3.0.7, Kistler Group, Winterthur, Switzerland) was used for analysis of each delivery bowled. All kinetic variables were normalized to body weight (BW). The continuous force-time curve in the vertical and anterior/posterior axes of the entire FFC was also assessed for each delivery. As previously stated, a customized MATLAB script was used to time normalize the data to 100% of the stance phase with the only variation being that force-time curves in both the vertical and anterior/posterior axes were normalized to the entire FFC phase, and not just to ball release.

### 2.6. Statistical Analyses

Descriptive statistics (mean ± standard deviation) were used to profile each measured parameter. Normality of data was assessed by visual analysis of the Q-Q plots [28,29]. To assess the reliability of all discrete variables, intra-class correlation coefficient (ICC) and coefficient of variance (CV) were determined. An ICC ≥ 0.70 [30] and a CV ≤ 10% was deemed acceptable [31]. A two-tailed paired-samples *t*-test was used to determine significant changes in the mean of the first and last three overs for each discrete variable within delivery lengths. Due to this statistical approach and to decrease the chances of making a Type 1 error, the criterion for significance was set at *p* ≤ 0.01 [32]. Hedges’ *g* effect sizes and 95% confidence intervals were also calculated for the first and last three over comparisons. The magnitude of effect was assessed on the following scale: Less than 0.2 was considered a trivial effect, 0.2 to 0.49 a small effect, 0.5 to 0.79 a medium effect, and greater than 0.8 a large effect [33]. These statistics were computed using the Statistics Package for Social Sciences Version 23.0 (IBM, Armonk, NY, USA).

Statistical parametric mapping (SPM) was used to evaluate if significant changes in kinematics and kinetics occurred at points other than the commonly assessed peaks and other discrete measures. Briefly, SPM uses random field theory to objectively identify field regions which co-vary significantly with the experimental design [34,35]. Two-tailed paired sample *t*-tests were performed on the normalized time series data during FFC to determine if a significant (*p* < 0.05) change occurred between the first and last three overs of the testing protocol, with respect to the front knee kinematics and vertical and anterior/posterior axes GRF curves. The SPM analysis required four steps as outlined in De Ridder, et al. [36]. All SPM analyses were implemented in MATLAB R2015b (The MathWorks Inc, Natick, MA, USA) using the open source package “rft1d” located at http://www.spm1d.org/ [37].

## 3. Results

**Figure 2:**
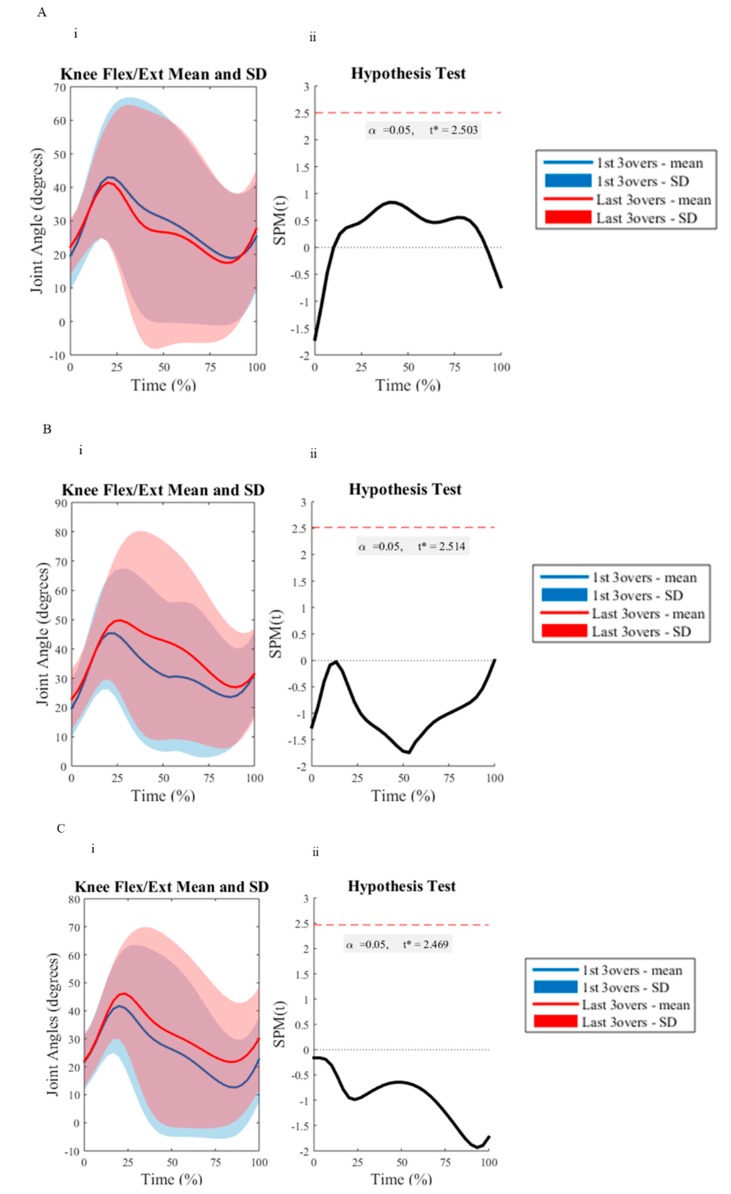
The sagittal plane (flexion/extension) knee joint angle trajectories between the first three (blue line) and last three (red line) of the eight over-spell, for the short (**A**), good (**B**), and full (**C**) delivery lengths. (**i**) is the mean knee joint angle trajectories with standard deviation clouds (first three overs = blue, last three overs = red). (ii) displays the paired samples SPM{t}: The *t* statistic as a function of time, describing the strength and slope of the relationship between the first three overs and last three overs testing measures. The dotted horizontal line indicates the random field theory thresholds for significance, and *p* values indicate the likelihood that a random process of the temporal smoothness would be expected to produce a suprathreshold cluster of the observed size.

**Figure 3:**
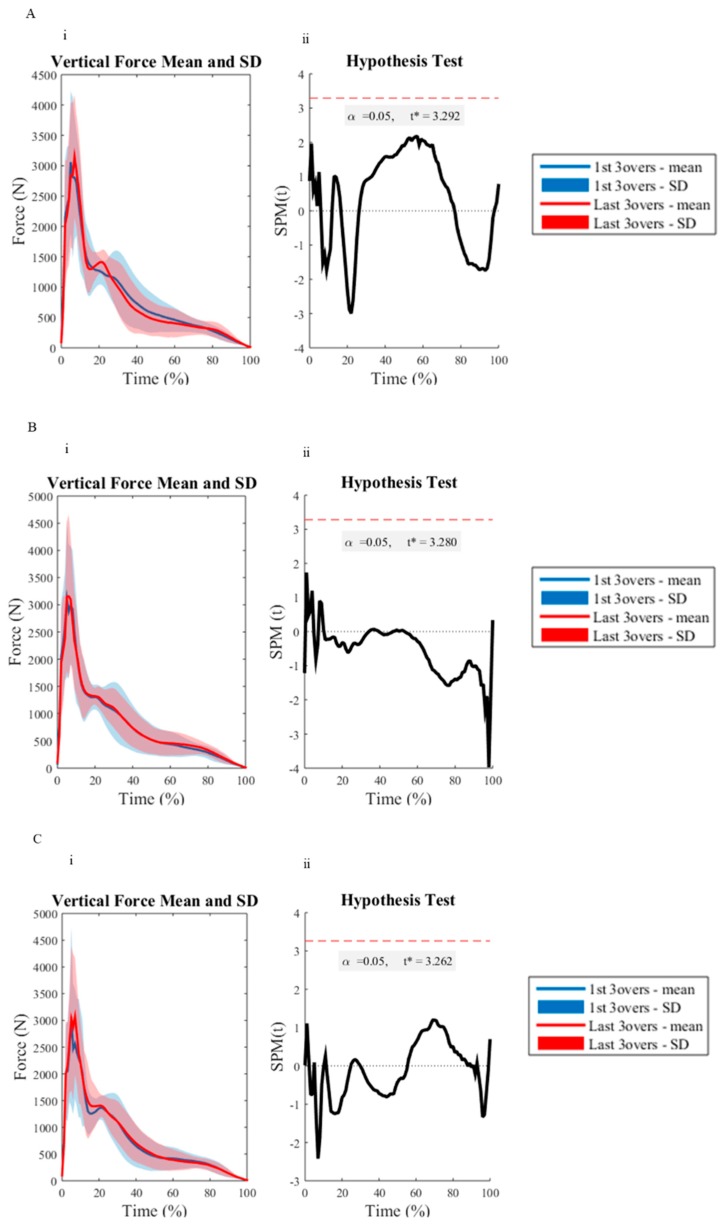
The vertical ground reaction force trajectories between the first three (blue line) and last three (red line) of an eight over-spell, for the short (**A**), good (**B**), and full (**C**) delivery lengths. (**i**), is the mean ground reaction force trajectory with standard deviation clouds (first three overs = blue, last three overs = red). (**ii**), displays the paired samples SPM{t}: The *t* statistic as a function of time, describing the strength and slope of the relationship between the first three overs and last three overs testing measures. The dotted horizontal line indicates the random field theory thresholds for significance, and *p* values indicate the likelihood that a random process of the temporal smoothness would be expected to produce a suprathreshold cluster of the observed size.

**Figure 4:**
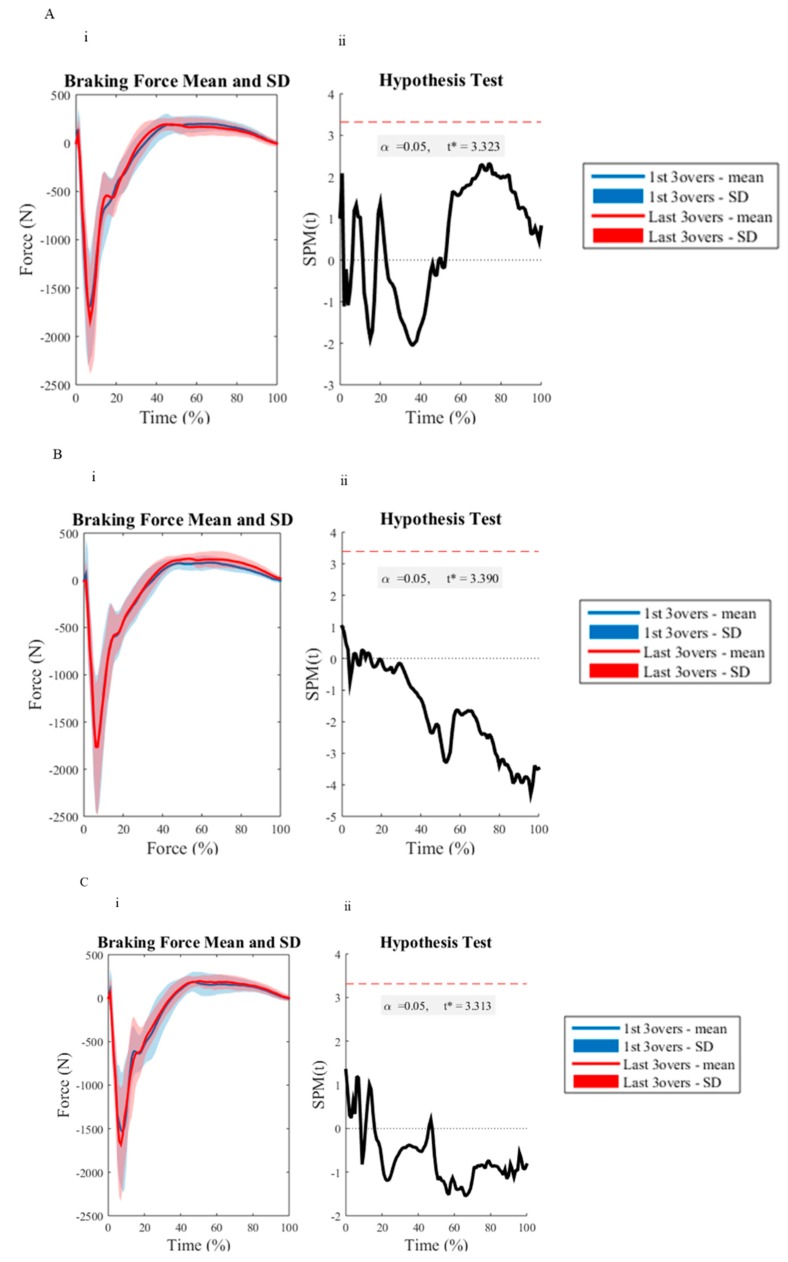
The braking (horizontal) ground reaction force trajectories between the first three (blue line) and last three (red line) of an eight over-spell, for the short (**A**), good (**B**), and full (**C**) delivery lengths. (**i**), is the mean ground reaction force trajectory with standard deviation clouds (first three overs = blue, last three overs = red). (**ii**), displays the paired samples SPM{t}: The *t* statistic as a function of time, describing the strength and slope of the relationship between the first three overs and last three overs testing measures. The dotted horizontal line indicates the random field theory thresholds for significance, and *p* values indicate the likelihood that a random process of the temporal smoothness would be expected to produce a suprathreshold cluster of the observed size.

All investigated variables were deemed to be normally distributed as determined by the Q-Q plot analysis. All discrete reliability measures (ICC = 0.72–0.99, CV = 1.46–9.70%) were deemed acceptable, except for the vertical loading rate measure for the first (CV = 19.39–20.97%) and last (CV = 10.33–20.08%) three overs for all delivery lengths. There were no significant differences between BRS between the first and last three overs with respect to the measured delivery lengths (Table 1). Table 2 and Table 3 outline the kinematic and BW normalized kinetic results measured during the first and last three overs of the testing protocol, respectively. There were no significant differences between the assessed variables across the short, good and full delivery lengths. However, there was a 14% increase in horizontal run-up velocity for the short delivery length from the first to last three overs of the testing protocol, which had a medium effect. However, this was non-significant. Figure 2, Figure 3 and Figure 4 depict the differences between the first and last three overs of an eight over bowling spell, as assessed using SPM upon front knee kinematics, and vertical and horizontal GRF during FFC, respectively. The SPM curve analysis revealed no significant differences.

## 4. Discussion

This is the first study to investigate the GRF experienced during FFC, in addition to bowling kinematics, for an eight over bowling spell with respect to different delivery lengths among pace bowlers. The results reinforced previous research [3,4,5,6,8], as the pace bowlers in this study were able to maintain their technique and BRS throughout a single extended bowling spell. The ability to maintain similar biomechanics was contrary to the study hypothesis and may suggest that pace bowlers are able to tolerate the repeatedly high whole-body biomechanical load (as determined by GRF) experienced within an eight over spell within the joints assessed. The results from this study provide valuable information about the loading experienced by a pace bowler during an eight over spell, as well as providing support for current bowling load monitoring practices of counting the total number of deliveries bowled.

The ability to maintain a high BRS throughout an extended bowling spell could reduce the decision and stroke execution time for an opposing batter. In accordance with the study hypothesis, there was no significant difference in BRS between the first three and last three overs of an eight over spell. The lack of significant change in BRS with trivial to small effect, is similar to previous extended bowling spell investigations which found no significant changes among elite (spell 1 = 34.92 ± 1.41 m·s^−1^, spell 2 = 34.83 ± 1.25 m·s^−1^) [8] and state first grade and high-performance pace bowlers (2nd over = 31.73 ± 0.78 m·s^−1^, 8th over = 31.93 ± 0.29 m·s^−1^) [3]. The ability to maintain BRS throughout an extended bowling spell is beneficial to pace bowling performance, as it should reduce the decision-making time for batters.

With respect to different delivery lengths among pace bowlers, there was no significant change in the assessed kinematics between the first and last three overs of an eight over spell. There was, however, a 14%, medium effect size increase in horizontal run-up velocity for the short delivery length in the final three overs when compared to the first three overs of the eight over spell. This result supported the findings of Burnett, Elliott, and Marshall [7], who documented a non-significant increase (0.2 m·s^−1^) in run-up velocity, although different delivery lengths were not assessed. Burnett, Elliott, and Marshall [7] suggested that increases in approach speed during the later stages of an extended bowling spell may be required to counter any changes in other areas (e.g., trunk and knee kinematics) of the pace bowling system. Regardless, the current results showed there were no significant changes in bowling kinematics within any of the delivery lengths, and it should be noted that the data from the current investigation can only speculate that an increase in run-up velocity was in an effort to use momentum to maintain the kinematics displayed at FFC. As such a hypothesis would require an extended analysis of the system energy flow.

The results from this study also indicated no significant differences in GRF during FFC throughout an eight over spell, regardless of delivery length. The pace bowlers within this study were able to maintain appropriate GRF during FFC to ensure the maintenance of BRS for each delivery length. Therefore, it appears that the bowlers within the current study were able to tolerate the repeated bouts of eccentric contractions associated with the high GRF experienced during FFC of the delivery stride [14]. However, it is important to note that while it is not uncommon for pace bowlers to perform an eight over spell [3], they may also be required to bowl up to 50 overs (300 deliveries or more) during a multi-day match depending on the match circumstances and game strategy [38]. Although GRF may not change during an eight over spell, there is a need for future research to assess the loading experienced by a pace bowler within an entire multi-day match, where multiple extended bowling spells will be completed.

The results from the SPM revealed no significant difference for the vector field analyzed GRF or front knee kinematics during FFC, regardless of the delivery length. These findings are in accordance with the discrete GRF and kinematic measures and further suggest that pace bowlers can appropriately maintain their technique in the joints assessed throughout a single eight over spell for each delivery length. It is also noteworthy that the use of SPM provided valuable new insight into the whole-body biomechanical load experienced in the vertical and braking/propulsive planes and sagittal knee kinematics by pace bowlers. The use of SPM visually presents the average GRF and knee kinematic traces of all pace bowlers, providing critical information about the loading and movement patterns present during FFC, not just the commonly assessed peaks or average values as suggested within previous research [39,40]. The use of SPM may allow future investigations to evaluate specific portions of FFC that may be most associated with specific alterations as a result of multiple extended bowling spells.

The consistent GRF reported throughout the eight over spell is of practical importance when considering current bowling load monitoring practices. Current practices stipulate that the total number of deliveries bowled, irrespective of the length of spell or delivery, be used as a tool to monitor bowling load [15,18,41]. The study findings provide support for current bowling load monitoring practices and suggest that strength and conditioning coaches do not need to track or prescribe bowling loads with respect to the length of spell or delivery for up to eight overs. However, it is important to note that while the results of the current study highlight consistency in whole-body external biomechanical load during FFC of the delivery stride throughout and extended eight-over spell and changes in delivery length, there is a need for greater research into the individual variations between bowlers. This is with respect to how joint-based load is distributed throughout the system when performing extended bowling spells which target different delivery lengths. As such, individuals with similar whole-body measures of load may be using different joint based strategies during performance that could have implications upon performance or injury which are not detailed by whole-body measures of load.

There are certain limitations for this study. The participant numbers utilized in this study were low (n = 9), although they were similar to previous research analyzing the effects of an extended bowling spell upon biomechanics and BRS in pace bowlers [7,8,42]. The full-body model utilized for kinematic data analysis involved an exported virtual marker set that is calculated instead of direct measured marker location. However, the estimated marker locations and subsequent model produced has previously been found to be a reliable and valid measure of joint kinematics during dynamic movements when in combination with a biomechanical model [19,20,21]. Furthermore, the model used within the current investigation was based upon previously published models for cricket pace bowlers [11,22], ultimately these factors indicate that the developed model was appropriate for the determination of joint kinematics. Therefore, as with interpretation of kinematic data, interpretation of the magnitude of effect and not just statistical significance is critical to interpretation of a meaningful or clinically relevant magnitude of change or difference. The use of laboratory-based testing may limit the ecological validity of the study. However, laboratory testing allowed for a more detailed analysis of the pace bowling action and has previously been used within the literature [3,7,8,42]. Future research should attempt to investigate the biomechanics of pace bowlers during an actual multi-day match or evaluate changes in timing or segmental coordination leading to FFC that could mediate in an effort to maintain the variables assessed in this study. Finally, the current investigation did not take an individual approach to understanding the variability and implications of an extended bowling spell within each pace bowler. However, the focus of the investigation was on the global characteristics which may alter as a result of an extended bowling spell, regardless of technique. Within the context of these limitations, this study still provides valuable insight into the loading experienced by a pace bowler throughout a single extended bowling spell, while also adding support to current bowling load monitoring practices.

## 5. Conclusions

In conclusion, an eight over spell targeting short, good and full delivery lengths, does not result in significant changes to a pace bowler’s biomechanics or BRS. The results suggest that pace bowlers can tolerate the repeated high whole-body external biomechanical loading (as represented by GRF) experienced during FFC without any one isolated variable significantly changing technique. As GRF was maintained throughout the bowling spell, this provides support for current bowling load monitoring practices used in the applied setting. Ultimately, this research indicates that within an eight over spell, pace bowlers are able to maintain BRS and technique while employing a range of delivery lengths in an attempt to dismiss opposing batters. Additional research is required to determine the effects of repeated bowling spells upon the biomechanics and BRS of pace bowlers within the multi-day match format.

## Figures and Tables

**Table 1:** The first and last three over (mean ± standard deviation) ball release velocity (BRS) for the short, good and full-length deliveries in cricket pace bowlers (n = 9).

Delivery Length	First Three over BRS (m·s^−1^)	Last Three over BRS (m·s^−1^)	*p*	*g* (95% CI)	Description
Short	29.55 ± 1.61	29.22 ± 1.33	0.11	0.21 (−0.47–0.90)	Small
Good	29.68 ± 1.74	29.50 ± 1.39	0.29	0.11 (−0.62–0.84)	Trivial
Full	29.57 ± 1.69	29.23 ± 1.31	0.44	0.21 (−0.48–0.91)	Small

m·s^−1^ = meters per second, *p* = *p* value, *g* = Hedges’ *g* effect size, 95% CI = 95% confidence intervals.

**Table 2:** The horizontal run-up velocity, knee angle at front foot contact (FFC) and ball release (BR), trunk flexion from FFC to BR, and shoulder angle at BR, between the first and last three overs of the testing protocol for the short, good, and full delivery lengths (n = 9).

Variable	Delivery Length	First Three overs	Last Three overs	*p*	*g* (95% CI)	Descriptor
Horizontal run-up velocity (m·s^−1^)	Short	3.80 ± 0.77	4.35 ± 0.67	0.29	−0.73 (−1.06–−0.39)	Medium
	Good	3.79 ± 1.03	4.19 ± 0.61	0.62	−0.45 (−0.84–−0.06)	Small
	Full	4.05 ± 0.71	4.16 ± 0.68	0.06	−0.15 (−0.47–0.17)	Trivial
Knee angle at FFC (°)	Short	21.84 ± 10.07	23.29 ± 8.14	0.30	−0.15 (−4.38–4.08)	Trivial
	Good	21.78 ± 9.88	22.34 ± 9.29	0.72	−0.06 (−4.49–4.37)	Trivial
	Full	23.04 ± 9.40	22.65 ± 8.43	0.76	0.04 (−4.08–4.17)	Trivial
Knee Angle at BR (°)	Short	40.87 ± 24.82	43.00 ± 29.35	0.62	−0.08 (−12.63–12.5)	Trivial
	Good	41.65 ± 29.18	45.20 ± 28.76	0.13	−0.12 (−13.5–13.27)	Trivial
	Full	43.59 ± 27.95	46.17 ± 26.16	0.26	−0.01 (−12.6–12.42)	Trivial
Trunk flexion from FFC–BR (°)	Short	35.49 ± 21.26	36.49 ± 18.73	0.71	−0.05 (−9.3–9.21)	Trivial
	Good	37.48 ± 19.77	37.02 ± 17.85	0.78	0.02 (−8.68–8.72)	Trivial
	Full	36.56 ± 18.23	36.79 ± 18.07	0.76	−0.01 (−8.4–8.37)	Trivial
Shoulder Angle at BR (°)	Short	178.00 ± 16.42	178.74 ± 22.20	0.55	−0.03 (−9.01–8.98)	Trivial
	Good	172.21 ± 16.73	180.37 ± 19.37	0.05	−0.43 (−8.79–7.93)	Small
	Full	176.05 ± 16.92	178.41 ± 20.44	0.46	−0.12 (−8.79–8.55)	Trivial

m·s^−1^ = meters per second, ° = degrees, *p* = *p* value, *g* = Hedges’ *g* effect size; 95% CI = 95% confidence intervals.

**Table 3:** The body weight normalized peak vertical force (PVF), peak braking force (PBF), vertical impulse, braking impulse, average (Avg) vertical loading rate (VLR), and average braking loading rate (BLR) loading rate between the first and last three overs for the short, good, and full delivery lengths among cricket pace bowlers (n = 9).

Variable	Delivery Length	First Three overs	Last Three overs	*p*	*g* (95% CI)	Descriptor
PVF (N·BW^−1^)	Short	5.31 ± 1.49	5.49 ± 1.38	0.24	−0.12 (−0.78–0.54)	Trivial
	Good	5.47 ± 1.48	5.47 ± 1.32	0.98	0.00 (−0.65–0.65)	Trivial
	Full	5.53 ± 1.34	5.54 ± 1.29	0.94	−0.01 (−0.62–0.6)	Trivial
PBF (N·BW^−1^)	Short	−2.85 ± 0.62	−2.82 ± 0.63	0.73	−0.05 (−0.33–0.24)	Trivial
	Good	−3.07 ± 0.61	−2.94 ± 0.6	0.39	−0.21 (−0.48–0.08)	Small
	Full	−2.91 ± 0.46	−2.93 ± 0.58	0.89	0.04 (−0.21–0.28)	Trivial
Vertical impulse (N.s·BW^−1^)	Short	0.28 ± 0.03	0.28 ± 0.03	0.71	0.00	Trivial
	Good	0.28 ± 0.03	0.28 ± 0.03	0.98	0.00	Trivial
	Full	0.28 ± 0.02	0.28 ± 0.03	0.26	0.00 (−0.01–0.01)	Trivial
Braking impulse (N.s·BW^−1^)	Short	−0.12 ± 0.02	−0.11 ± 0.02	0.17	−0.48 (−0.49–−0.47)	Small
	Good	−0.12 ± 0.02	−0.11 ± 0.01	0.14	−0.6 (−0.61–−0.60)	Moderate
	Full	−0.12 ± 0.02	−0.12 ± 0.01	0.50	0.00 (−0.01–0.01)	Trivial
Avg VLR (N.s^−1^·BW^−1^)	Short	233.41 ± 119.06	252.58 ± 109.37			
	Good	249.52 ± 122.64	224.51 ± 80.48			
	Full	254.26 ± 112.78	255.91 ± 93.64			
Avg BLR (N.s^−1^·BW^−1^)	Short	−85.31 ± 29.68	−83.31 ± 25.67	0.62	−0.07 (−12.9–12.75)	Trivial
	Good	−91.9 ± 30.97	−87.48± 29.33	0.29	−0.14 (−14.07–13.8)	Trivial
	Full	−86.83 ± 23.49	−88.28 ± 31.21	0.69	0.05 (−12.71–12.8)	Trivial

N·BW^−1^ = newtons per body weight, N.s·BW^−1^= newton second per body weight, N.s^−1^·BW^−1^ = newtons per second per body weight, *p* = *p* value, *g* = Hedges’ *g* effect size, 95% CI = 95% confidence intervals. Italicized variables are presented for information purpose but did not have the level of reliability deemed acceptable.

## Abbreviations

The following abbreviations are used in this manuscript:

BRS	Ball release speed
GRF	Ground reaction force
FFC	Front foot contact
m	Meter
kg	Kilogram
3D	Three-dimensional
Hz	Hertz
BW	Body weight
ICC	Intra-class correlation coefficients
CV	coefficient of variance
SPM	Statistical parametrical mapping
m·s^−1^	Meters per second
95% CI	95% confidence intervals
BR	Ball release
°	Degree
PVF	Peak vertical force
PBF	Peak braking force
Avg	Average
VLR	Vertical loading rate
BLR	Braking loading rate
N·BW^−1^	Newtons per body weight
N.s·BW^−1^	Newton seconds per body weight
N.s^−1^·BW^−1^	Newtons per second per body weight
